# Antibodies to Crucial Epitopes on HSV-2 Glycoprotein D as a Guide to Dosing an mRNA Genital Herpes Vaccine

**DOI:** 10.3390/v14030540

**Published:** 2022-03-05

**Authors:** Lauren M. Hook, Sita Awasthi, Tina M. Cairns, Mohamad-Gabriel Alameh, Bernard T. Fowler, Kevin P. Egan, Molly M. H. Sung, Drew Weissman, Gary H. Cohen, Harvey M. Friedman

**Affiliations:** 1 Infectious Disease Division, Department of Medicine, Perelman School of Medicine, University of Pennsylvania, Philadelphia, PA 19104, USA; lhook@pennmedicine.upenn.edu (L.M.H.); sawasthi@pennmedicine.upenn.edu (S.A.); mg.alameh@pennmedicine.upenn.edu (M.-G.A.); bernard.fowler@pennmedicine.upenn.edu (B.T.F.); kevinpe@pennmedicine.upenn.edu (K.P.E.); dreww@pennmedicine.upenn.edu (D.W.); 2 Department of Basic and Translational Sciences, School of Dental Medicine, University of Pennsylvania, Philadelphia, PA 19104, USA; tmcairns@upenn.edu (T.M.C.); ghc@upenn.edu (G.H.C.); 3 Acuitas Therapeutics Inc., Vancouver, BC V6T 1Z3, Canada; msung@acuitastx.com

**Keywords:** herpes simplex virus type 2, nucleoside-modified mRNA, lipid nanoparticle, glycoprotein D, genital herpes vaccine, IgG ELISA, neutralizing antibodies, epitope mapping, surface plasmon resonance

## Abstract

The toxicity of mRNA-lipid nanoparticle (LNP) vaccines depends on the total mRNA-LNP dose. We established that the maximum tolerated dose of our trivalent mRNA-LNP genital herpes vaccine was 10 μg/immunization in mice. We then evaluated one of the mRNAs, gD2 mRNA-LNP, to determine how much of the 10 μg total dose to assign to this immunogen. We immunized mice with 0.3, 1.0, 3.0, or 10 μg of gD2 mRNA-LNP and measured serum IgG ELISA, neutralizing antibodies, and antibodies to six crucial gD2 epitopes involved in virus entry and spread. Antibodies to crucial gD2 epitopes peaked at 1 μg, while ELISA and neutralizing titers continued to increase at higher doses. The epitope results suggested no immunologic benefit above 1 μg of gD2 mRNA-LNP, while ELISA and neutralizing titers indicated higher doses may be useful. We challenged the gD2 mRNA-immunized mice intravaginally with HSV-2. The 1-μg dose provided total protection, confirming the epitope studies, and supported assigning less than one-third of the trivalent vaccine maximum dose of 10 μg to gD2 mRNA-LNP. Epitope mapping as performed in mice can also be accomplished in phase 1 human trials to help select the optimum dose of each immunogen in a multivalent vaccine.

## 1. Introduction

Nucleoside-modified mRNA-lipid nanoparticle (LNP) vaccines have been highly successful in reducing hospitalizations and deaths from COVID-19 [1]. mRNA vaccines for rabies, influenza, and cytomegalovirus are in human trials (https://clinicaltrials.gov/ct2/show/NCT05085366, accessed on 30 January 2022) [2,3]. Other viral vaccines are likely to follow, possibly including our candidate HSV-2 trivalent vaccine to prevent genital herpes [4,5,6,7].

The mRNA-LNP vaccines for COVID-19 demonstrated dose-dependent toxicity in phase 1 human studies [8,9]. An mRNA dose of 30 µg of the Pfizer/BioNTech COVID-19 vaccine was well tolerated, however higher doses had more adverse reactions [9]. The cutoff dose for the Moderna vaccine was 100 µg [9]. These vaccines use different proprietary LNP formulations. The LNP component tends to be the reactogenic constituent in the vaccine, and the LNP content increases proportional to the mRNA dose [10]. The COVID-19 mRNA-LNP vaccine contains a single mRNA encoding the Spike protein [1]. Our genital herpes vaccine contains three mRNAs while the CMV vaccine in phase 3 human trials contains 6 mRNAs [4,5]. Multivalent vaccines need to use lower doses of individual immunogens to keep the total mRNA-LNP content below toxic levels.

Antigen doses for vaccines are often determined in dose escalation phase 1 human trials based on balancing toxicity and immunogenicity. The selected dose is then evaluated for efficacy in much larger and more costly phase 2 and 3 human trials. Here, we evaluated whether measuring antibody responses to crucial epitopes on immunogens in dose escalation (phase 1-like) studies adds value to more established methods, such as serum IgG ELISA and neutralizing antibody assays, in selecting the optimum dose of immunogens to include in larger efficacy studies. We used the mouse model of genital herpes and gD2 mRNA-LNP as the test immunogen to evaluate our hypothesis that epitope mapping will help select the optimal dose of an immunogen to include in a multivalent vaccine.

We previously performed epitope mapping studies using high throughput biosensor technology to measure antibody responses to crucial epitopes on HSV-2 glycoprotein D (gD2) in immunized mice, guinea pigs, and humans [5,11,12,13]. In the guinea pig HSV-2 genital infection model, antibody responses to crucial gD2 epitopes correlated with vaccine efficacy [11]. The greater the number of crucial gD2 epitopes recognized by the immune serum and the higher the antibody titer, the greater the protection was against the genital disease [11]. Here, we compare the utility of epitope mapping with serum IgG ELISA and neutralizing antibody titers for selecting the lowest effective dose of an mRNA immunogen to include in a multivalent vaccine.

## 2. Materials and Methods

### 2.1. Evaluating Trivalent mRNA-LNP Vaccine Toxicity

For toxicity studies, female BALB/c mice (Charles River) age 7–9 weeks were immunized twice 28 days apart intramuscularly (IM) in the hind limb hip muscle with a total dose of 1, 3, or 10 µg in 30 µL containing equal concentrations of gC2, gD2, and gE2 mRNA-LNP, or with sterile saline as a control [5]. The DNA constructs designed to prepare the mRNA and procedures used to generate the mRNA have been described previously [5]. The mRNA was encapsulated in LNP prepared by Acuitas [5]. Mice were evaluated for weight loss and hind limb mobility daily for 6–7 days after the first and second immunizations. Mice received a daily score of 2 for a moderate reduction in hind limb mobility, 1 for a minor reduction, and 0 for no reduction.

### 2.2. Immunizing with gD2 mRNA-LNP

To determine the lowest effective dose of gD2 mRNA to include in the vaccine, female BALB/c mice were immunized twice at 28-day intervals with gD2 mRNA-LNP at doses of 0.3, 1.0, 3.0, or 10 µg (10 mice/group) diluted in 30 µL of sterile saline. An additional group of 10 mice received two immunizations with 10 µg of Poly(C) RNA-LNP as a control. Serum was obtained just prior to the first immunization and four weeks after the first and second immunizations and stored at –80 °C.

### 2.3. Serum IgG ELISA and Neutralizing Antibody Titers

Purified baculovirus-derived gD2 was added to 96-well High Binding Costar microtiter plates at 100 ng/well in 50 mM sodium bicarbonate pH 8.5 binding buffer, incubated for 1 h at room temperature then overnight at 4 °C, and blocked for 2 h with 5% (wt/vol) nonfat milk in PBS 0.05% Tween 20 [14]. Serial 2-fold dilutions of serum starting at 1:1000 were added to gD2-coated wells. Bound IgG was detected at an optical density of 405 nm using horseradish peroxidase-conjugated anti-mouse IgG at a 1:2000 dilution followed by 2,2′-azino-bis-3-ethylbenzothiazoline-6-sulfonic acid (ABTS). Endpoint titers were determined by regression analysis as the serum dilution resulting in an OD reading two-fold above background.

Neutralizing titers were measured by incubating 2-fold serial dilutions of mouse sera with 5% human serum as the source of complement obtained from an HSV-1- and HSV-2 seronegative donor and 100 PFU of HSV-2 at 37 °C for 1 h. The remaining virus was determined by plaque assay on Vero cells. The neutralizing titer was defined as the highest serum dilution that reduced plaques by ≥50% [14].

### 2.4. Biosensor-Based Antibody Competition Assay

Antibody competition assays were performed using the Carterra Microfluidics continuous flow microspotter surface plasmon resonance imaging (CFM/SPRi, Salt Lake City, UT, USA) system [5,11,15]. Monoclonal antibodies (MAb) that recognize gD were amine-coupled to a CDM200M sensor chip (XanTec GmbH, Dusseldorf, Germany). Antibody competition assays were then performed in the surface plasmon resonance imager (IBIS MX96, Salt Lake City, UT, USA) in a pre-mix assay format by saturating soluble gD2(285t) at a concentration of 75 ng/reaction with 2% (1:50 dilution) mouse sera. Each gD2(285t)/mouse serum mix was flowed across the sensor chip spotted with the gD MAbs. gD2 alone was flowed across the chip for the first 2–3 cycles and every 10th cycle to establish the background binding of gD2 compared to gD2 plus mouse serum. Data were recorded as response units (RU). The blocking activity of mouse sera was calculated for each Mab as a percentage using the formula: [1 − (RU gD2(285t) + mouse serum)/(RU gD(285t) alone)] × 100. Occasionally, the (RU gD2(285t) + mouse serum) yielded a negative RU value that was lower than the baseline value of buffer alone, resulting in percent blocking values that exceeded 100%.

### 2.5. HSV-2 Vaginal Infection and Vaccine Efficacy

Mice were injected subcutaneously with 2 mg of medroxyprogesterone five days prior to infection [16]. The vagina was cleared using a sterile swab moistened with phosphate-buffered saline (PBS), and mice were infected intravaginally five weeks after the second immunization with 5 × 10^3^ PFU (275 LD_50_) HSV-2 strain MS. Mice were monitored for survival, weight loss, genital disease, and vaginal virus titers on days two and four post-infection, and HSV-2 DNA in dorsal root ganglia (DRG) at the time of humane euthanasia or at the end of the experiment on days 28–32 post-infection. Mice received a daily score of one each for redness/erythema, hair loss, vaginal exudate, and necrosis for a maximum daily score of four for genital disease.

### 2.6. Vaginal Virus Titers

Vaginal swabs were obtained on days two and four post-infection and placed in 1 mL of complete Dulbecco’s modified Eagle’s medium supplemented with L-glutamine, HEPES, antibiotics, and 5% fetal bovine serum. Serial 10-fold dilutions were evaluated by plaque assay on Vero cells [5].

### 2.7. qPCR for HSV-2 DNA Present in the DRG

DRG samples were analyzed in duplicate for HSV-2 genomic DNA [5]. Separate reactions were used to amplify HSV-2 DNA and the mouse adipsin gene. Five microliters of purified DNA (QiaCube HT, Germantown, MD, USA) were amplified via Taqman qPCR (Roche LightCycler 96, Indianapolis, IN, USA). The primers and probe used to amplify the HSV-2 Us9 gene were: Forward, GGCAGAAGCCTACTACTCGGAAAA; Reverse, CCATGCGCACGAGGAAA; and Probe, FAM-CGAGGCCGCCAAC-MGBNFQ. The primers and probe used to amplify the mouse adipsin gene were: Forward, GCAGTCGAAGGTGTGGTTACG; Reverse, GGTATAGACGCCCGGCTTTT; and Probe, FAM-CTGTGGCAATGGC-MGBNFQ. The DRG HSV-2 DNA copy number was expressed as log_10_ DNA copies per 10^5^ adipsin genes. Samples with less than one copy by 40 cycles in duplicate wells were considered negative. If only one of the duplicate wells was positive, the sample was tested in triplicate. The DRG sample was considered positive for HSV-2 DNA if two or more of the triplicates were positive [4].

### 2.8. Statistical Analysis

Area under the curve (AUC) *p* values for weight loss and leg toxicity were calculated by the Mann–Whitney–Wilcoxon Test with Holm adjustment for multiple comparisons using R Studio Version 1.3.1056 with R software version 4.0.2 (R Core Team, 2020, DE, USA) and the trapezoid rule with function “AUC” from the package “DescTools” version 0.99.44. *p* values comparing endpoint serum IgG ELISA titers after the first and second immunization were calculated by Wilcoxon matched pairs signed rank test. The two-tailed Mann–Whitney test was used to compare gD2 IgG ELISA, neutralizing antibody titers, epitope antibody titers, day two and day four vaginal virus titers, and the HSV-2 DNA copy number in DRG. When multiple comparisons were performed, we used ordinary one-way ANOVA with Sidak’s correction. The log-rank (Mantel–Cox) test was used to calculate *p* values for survival. With the exception of AUC, *p* values were calculated using GraphPad Prism version 9.2.0. Results were considered significant at *p* < 0.05.

### 2.9. Study Approval

The mouse studies were approved by the University of Pennsylvania Institutional Animal Care and Use Committee under protocol 805187.

## 3. Results

### 3.1. Experimental Design and Rationale

The study design can be divided into three parts. In experiment 1, we immunized mice with our gC2, gD2, and gE2 trivalent mRNA-LNP vaccine to determine the total mRNA-LNP dose per administration that is safe. These results established the upper limits for the total dose of mRNA-LNPs, however did not define the optimum dose of the individual mRNA-LNP immunogens within that total. In experiment 2, we performed immunology assays as a guide for determining the optimum dose of an individual mRNA-LNP immunogen to include in a multivalent vaccine. We used only one of the immunogens, gD2 mRNA-LNP, to test our hypothesis that measuring antibody responses to crucial, functional epitopes on an antigen adds value to more standard assays, such as serum IgG ELISA and neutralizing antibodies, to define the optimum immunogen dose. Experiment 3 involved infecting the animals to assess vaccine efficacy. The goal was to determine which of the immunology assays best predicted the immunogen dose that protected the animals. Lessons learned about the predictive value of immune responses in preclinical studies will help select immunogen doses of multivalent vaccines in human trials.

### 3.2. Experiment 1: Toxicity Is Dose Dependent for the Trivalent mRNA-LNP Vaccine

**Figure 1:**
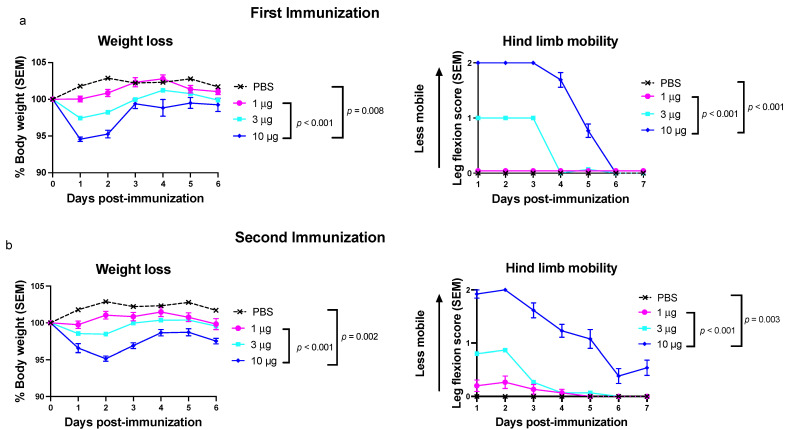
Trivalent mRNA-lipid nanoparticle (LNP) vaccine toxicity is dose dependent. Weight loss and hind limb mobility were assessed after (**a**) the first and (**b**) second immunization. The trivalent mRNA-LNP and phosphate-buffered saline (PBS) vaccination groups were weighed on days 1–6 post-infection and evaluated for mobility in the inoculated hind limb on days 1–7 post-immunization (weight: *n* = 15 animals in the 1-μg and 3-μg groups, 9 animals in the 10-μg group, and 5 animals in the PBS group; hind limb mobility: *n* = 15 mice in the 1-μg and 3-μg group, 13 mice in the 10-μg group, and 5 mice in the PBS group). *P* values were not calculated comparing 3 µg with PBS, 1 µg, or 10 µg. *p* values were determined by comparing area under the curve (AUC) using the Mann–Whitney–Wilcoxon test with Holm adjustment for multiple comparisons.

We evaluated vaccine toxicity in mice receiving the trivalent genital herpes vaccine that contains nucleoside-modified gC2, gD2, and gE2 mRNA packaged into LNPs at equivalent concentrations. The goal of experiment 1 was to determine the maximum tolerated dose when all three immunogens were included in the vaccine. BALB/c mice were immunized IM two times 28 days apart with 1, 3, or 10 µg of the trivalent HSV-2 mRNA-LNP vaccine or with sterile PBS as a control. Mice were evaluated after the first and second immunizations for weight loss and reduction in hind limb mobility. By comparing 10 µg of the trivalent mRNA-LNP vaccine with either PBS or the 1-µg dose, it was found that mice receiving the 10-µg dose lost more weight and developed greater loss in mobility of the immunized hind limb after both immunizations (Figure 1). Mice receiving the 3-µg dose had weight loss and decreased hind limb mobility in-between the 1-µg and 10-µg groups. We detected no differences for weight loss or hind limb mobility when comparing the 1-µg group with the PBS group or comparing the first with second immunization. Our mouse studies establish that lower doses of the trivalent mRNA vaccine are better tolerated than higher doses and support choosing the lowest vaccine dose possible that is effective. For the purposes of the current study, we established that the total dose of RNA-LNP immunogens within the vaccine should not exceed 10 µg.

### 3.3. Experiment 2: Dose Escalation Study of gD2 mRNA to Evaluate IgG ELISA Titers, Neutralizing Antibodies, and Antibodies to Crucial gD2 Epitopes

Choosing the lowest effective vaccine dose for each immunogen in a multivalent vaccine is challenging. We established in experiment 1 that the total dose of all three mRNA immunogens should not exceed 10 µg. The issue is whether to include equal concentrations of each mRNA immunogen or increase the concentration of one or more immunogens while decreasing another will improve the outcome without changing the total mRNA-LNP content. We evaluated gD2 mRNA as one of the three immunogens in the trivalent genital herpes vaccine to determine whether measuring antibody responses to crucial gD2 epitopes in a dose escalation study provides useful information beyond that gained from serum gD2 IgG ELISA and neutralizing antibody titers to establish the dose of an immunogen to include in a vaccine [11,15,17].

#### 3.3.1. Serum IgG ELISA Titers

**Figure 2:**
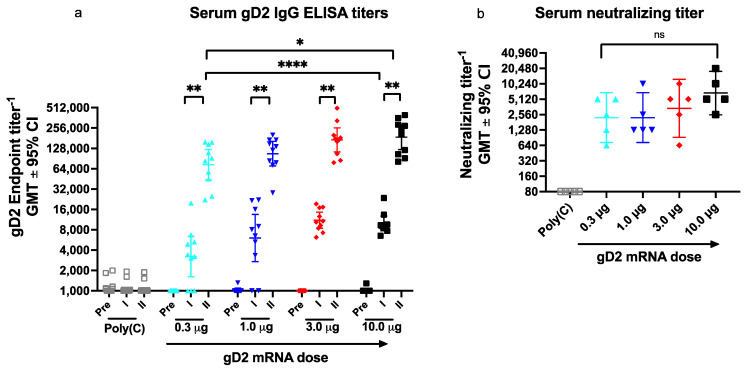
gD2 mRNA-LNP generates serum IgG ELISA and neutralizing antibody responses in a dose-dependent pattern. (**a**) Serum gD2 IgG ELISA titers were evaluated prior to the first immunization and four weeks after the first and second immunization (*n* = 10 sera/dose) (pre and prior to the first immunization; I and II, sera obtained after the first and second immunizations, respectively). (**b**) Neutralizing antibody titers were determined in the presence of complement (*n* = 5 sera/dose). *p* values comparing ELISA IgG titers after the first and second bleeds were calculated by the Wilcoxon matched pairs signed rank test. *p* values comparing different doses in (**a**,**b**) were calculated by the two-tailed Mann–Whitney test. *p* value in (**a**) comparing 10.0 μg with 0.3 μg was significantly different, however *p* values comparing 10.0 μg with 1.0 or 3.0 μg were not statistically different. * *p* < 0.05; ** *p* < 0.01; **** *p* < 0.0001. GMT, geometric mean titer, ns, not significant.

We first evaluated whether gD2 IgG ELISA is helpful when assessing the number of vaccine doses to administer. Mice were immunized twice at one-month intervals with gD2 mRNA-LNP at doses ranging from 0.3 μg to 10.0 μg (33-fold range). Sera were obtained prior to immunization and four weeks after the first and second immunizations. Serum gD2 IgG ELISA titers increased significantly after the second immunization at each gD2 mRNA dose (Figure 2a). Two immunizations at the lowest dose of 0.3 μg significantly exceeded the IgG ELISA titers after a single immunization with 10-μg gD2 (Figure 2a). These results indicate that a major boost in gD2 IgG ELISA titers occurs after the second immunization and demonstrate the value of serum IgG ELISA when selecting dosing frequency.

We next evaluated gD2 IgG ELISA titers after the second immunization to determine whether IgG ELISA is useful for selecting the optimal gD2 dose per administration of the vaccine. The serum IgG ELISA titers increased 2.4-fold when comparing 0.3 μg and 10.0 μg doses (Figure 2a). A dose-response pattern is apparent because higher gD2 doses produced higher ELISA titers, although titers changed relatively little over the 33-fold range. Based on the ELISA results, we would likely include gD2 mRNA-LNP at a dose of 3.33 μg in the trivalent vaccine, representing one-third of the maximum tolerated dose of 10 μg for all three immunogens. Our reasoning is that ELISA titers continued to rise with each higher dose, providing no clear guidance whether a dose lower than 3.33 μg would be equally efficacious.

#### 3.3.2. Serum Neutralizing Antibody Titers

We next evaluated neutralizing antibody responses after the second immunization using five sera per group that were selected based on the volume of serum available. We previously reported that neutralizing antibodies in guinea pigs immunized with the trivalent mRNA vaccine correlated with protection against genital infection [4]. Therefore, selecting the gD2 mRNA dose that produces the highest neutralizing titers without toxicity is important for the success of the trivalent mRNA vaccine. Neutralizing antibody titers at the three highest doses followed a dose-response pattern and were highest in the 10-μg group (Figure 2b). Therefore, we would likely choose a dose of 3.33 μg for gD2 mRNA in the trivalent vaccine for similar reasons as stated for ELISA titers.

#### 3.3.3. Antibodies to Crucial gD2 Epitopes

We used high throughput biosensor technology to define epitope-specific antibody responses to six crucial gD2 epitopes [5,11,12,13,15]. Antibodies that bind to these six gD2 epitopes perform one or more of the following functions: block gD2 binding to the herpesvirus entry mediator (HVEM) receptor, nectin-1 receptor, or both; block gD2 binding to the heterodimer fusion regulator, glycoproteins H/L (gH2/gL2); inhibit cell-to-cell spread; and alter the timing of important gD2 conformational changes [5,11,17].

We evaluated epitope-specific antibody responses to the six gD2 epitopes after the second immunization using five sera per gD2 dose that were selected based on the volume available. Our goal was to determine whether epitope mapping adds useful information to ELISA and neutralizing antibody titers during a phase 1-like dose escalation study to help select the optimum gD2 mRNA-LNP dose for phase 3 efficacy trials. Serum was mixed with purified gD2(285t) and then flowed sequentially over a biosensor chip spotted with multiple MAb [11,15]. Binding of gD2 to the MAb on the chip was measured for each gD2-immune serum mixture and compared to binding when gD2 was mixed with the buffer. A reduction in gD2 binding to a particular MAb when mixed with serum compared to buffer indicates competition and signifies that an antibody is present in the immune serum to an epitope recognized by the MAb on the chip.

**Figure 3:**
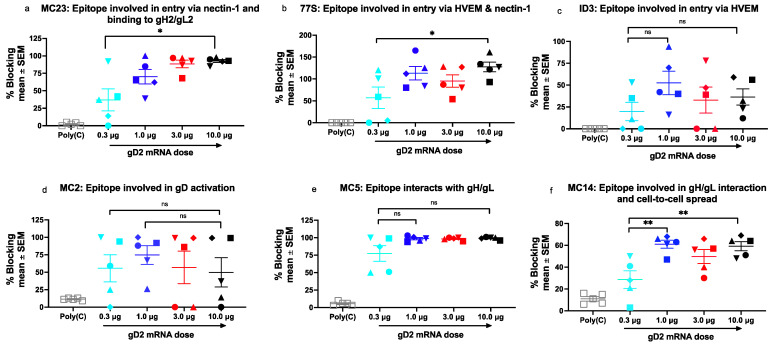
gD2 mRNA-LNP produces antibodies to important functional epitopes. Antibody responses were measured to six functional gD2 epitopes identified by MAb (**a**) MC23, (**b**) 77S, (**c**) ID3, (**d**) MC2, (**e**) MC5, and (**f**) MC14. Sera from five mice were evaluated per vaccine dose. The shape of the symbol identifies individual mice in the gD2 mRNA groups; for example, the animal indicated by a rectangle in (**a**) is also denoted by a rectangle in (**b**–**f**). We calculated *p* values comparing the highest dose (10 μg) with the lowest dose (0.3 μg), and the dose producing the highest antibody titer with the lowest antibody titer. *p* values were performed using the two-tailed Mann–Whitney test or ordinary one-way ANOVA with Sidak’s correction when multiple comparisons were performed. * *p* < 0.05; ** *p* < 0.01; ns, not significant.

MAbs that recognize identical or overlapping epitopes are referred to as a community of MAbs [13]. We spotted multiple MAbs from each community on the chip, and report the results of prototype MAbs for each community [11]. (1) Prototype MAb MC23 blocks gD2 binding to the nectin-1 receptor and to gH2/gL2 [11,15,17]. Titers of blocking antibodies to MC23 were highest in the 10-μg group and were significantly higher than in the 0.3-μg group that had the lowest titers (Figure 3a). (2) We next evaluated prototype MAb 77S, which recognizes an epitope that is involved with binding to HVEM and nectin-1 [11,15]. Similar to MC23, the antibody titers at 10 μg were highest and significantly greater than at 0.3 μg (Figure 3b). (3) We then assessed prototype MAb 1D3, which blocks gD2 binding to the HVEM receptor [11,15]. In contrast to the prior MAbs, the 10-μg group had lower antibody titers than at 1.0 μg, although differences did not achieve statistical significance (Figure 3c). This result suggests that at high doses, other (polyclonal) gD2 antibodies in the immune sera may alter ID3 epitope conformation or interfere with antibodies that bind to this epitope. We again detected the lowest antibody titers in the 0.3-μg group. (4) We next evaluated prototype MAbs MC2, which blocks gD2 activation thereby preventing its interaction with gH2/gL2 [17,18]. The antibody titers to the MC2 epitope followed a similar pattern as ID3 in that the 10-μg group had lower titers than the 1-μg group, suggesting possible changes to epitope conformation or interference by other antibodies in the sera (Figure 3d). (5) We then assessed prototype MAb MC5, which blocks gD2 binding to gH2/gL2 [18]. The antibody titers to MC5 plateaued at doses ≥ 1 μg (Figure 3e). (6) The final epitope was one that interacts with gH2/gL2 and mediates cell-to-cell spread and is recognized by prototype MAb MC14 [17,19]. As noted for several other epitopes, the 0.3-μg group had the lowest antibody titers (Figure 3f). We conclude that antibodies were produced to most epitopes at all immunization doses; that for some epitopes a high gD2 dose improved the antibody response, while for others, a high gD2 dose resulted in somewhat reduced antibody titers; and the 0.3-μg group generally produced the lowest antibody titers. No clear pattern emerged for choosing the optimal gD2 mRNA dose when analyzing antibody responses to individual epitopes.

**Figure 4:**
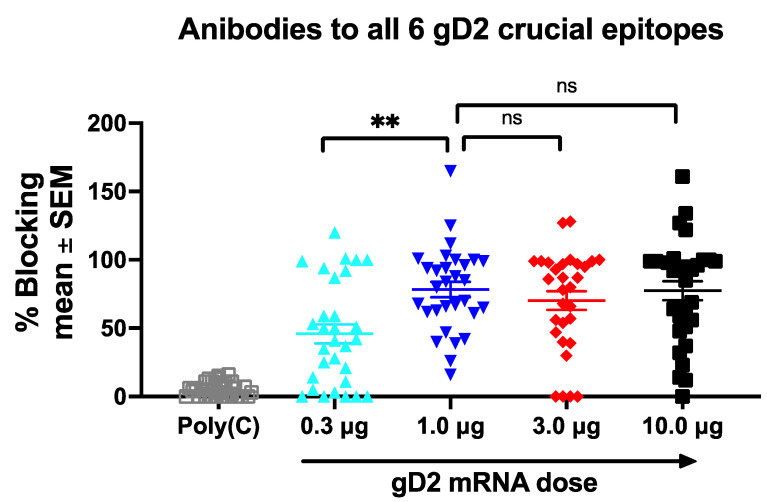
Combined responses to all six epitopes. The antibody titers to all six crucial gD2 epitopes at each immunization dose are displayed on a single graph. *p* values were calculated by ordinary one-way ANOVA with Sidak’s correction for multiple comparisons; *n* = 30 data points at each gD2 dose based on antibody responses to 6 epitopes x 5 sera; ** *p* < 0.01; ns, not significant.

Our prior studies indicated that high titer antibody responses to multiple epitopes are required for protection against genital HSV-2 infection with no individual epitope emerging as dominant [11]. Therefore, we combined the epitope-specific antibody responses into a single graph to demonstrate the polyclonal antibody response to all six epitopes at each gD2 dose (Figure 4). Importantly, we did not observe a dose-response pattern for antibody titers to gD2 epitopes. Instead, we identified a plateau in antibody titers at gD2 doses ≥1 μg with no further increase at higher doses. The plateau at 1 μg distinguishes the epitope antibody titers from IgG ELISA and neutralizing antibody responses that failed to plateau even at 10 μg. The epitope antibody titers suggest that the 1-μg dose is as effective as the higher doses in producing high titers of antibodies to the six crucial epitopes, leaving up to 9 μg available to accommodate the remaining two mRNA immunogens, if needed. In contrast, the IgG ELISA and neutralizing antibody titers supported using 3.33 μg without signaling that lower the gD2 mRNA dose may be equally effective.

### 3.4. Experiment 3: Vaccine Efficacy in gD2 mRNA Immunized Mice

**Figure 5:**
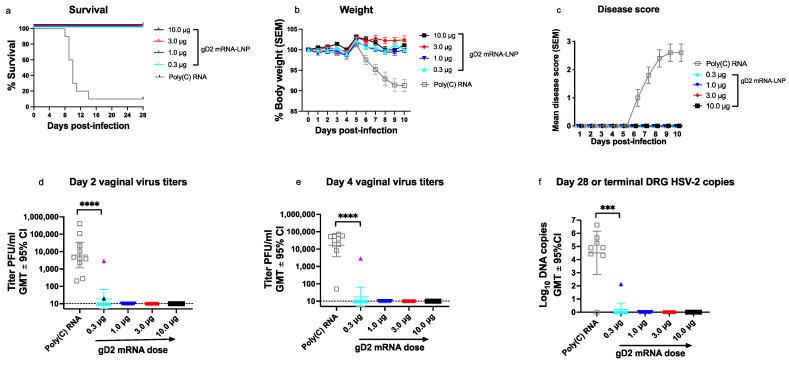
Vaccine efficacy in mice immunized with gD2 mRNA-LNP. (**a**) Survival curves: *p* < 0.0001 comparing the Poly(C) RNA group with all other groups. (**b**) Mean weight on days 1–10 post-infection. (**c**) Mean genital disease scores on days 1–10 post-infection. (**d**) Vaginal swab virus titers on day 2 post-infection (**e**) Vaginal swab virus titers on day 4 post-infection. The purple triangle symbol in (**d**,**e**) indicate that the vaginal virus titer is from the same animal. Dotted line in (**d**,**e**) indicates the assay limit of detection of 10 PFU/mL. (**f**) HSV-2 DNA copy number in dorsal root ganglia (DRG) at the time of euthanasia for the Poly(C) RNA group or at the end of the experiment on days 28–32 for the gD2 mRNA-LNP groups. The dark blue symbol identifies this animal as different from the positive animals in (**d**,**e**). *p* values in (**a**) were calculated by the log-rank (Mantel–Cox) test, and in (**d**–**f**) by the two-tailed Mann–Whitney test; *n* = 10 mice/group; ***, *p < 0.001;* ****, *p* < 0.0001.

To evaluate vaccine efficacy, the same mice used for immunology studies in Figure 2, Figure 3 and Figure 4 were challenged with HSV-2 at 5 × 10^3^ PFU (275 LD_50_) 35 days after the second immunization (10 mice/group). Nine of ten mice in the control (Poly(C) RNA) group required humane euthanasia, while all 40 gD2 mRNA-immunized animals survived, including those in the lowest dose group of 0.3 μg (Figure 5a). No animal immunized with gD2 mRNA lost weight (Figure 5b), developed genital disease (Figure 5c), or had any other sign of illness, including ruffled fur, hunched posture, abnormal gait, or lethargy.

We next evaluated mice for evidence of subclinical infection by measuring virus titers in vaginal swabs obtained on days two and four post-infection and by assaying for HSV-2 DNA copy number in DRG (site of latency) at the time of humane euthanasia or at the end of the experiment on days 28–32. All animals in the Poly(C) group had HSV-2 isolated from vaginal swabs on days two and four post-infection (Figure 5d,e). Only two of forty (5%) animals immunized with gD2 mRNA had positive vaginal virus cultures on day two and one of the same animals (2.5%) had a positive vaginal culture on day four (Figure 5d,e). Both animals with positive vaginal virus cultures were in the 0.3-μg group (Figure 5d,e). Six of seven animals in the Poly(C) group had HSV-2 DNA detected in DRG at the time of humane euthanasia 8–14 days post-infection (Figure 5f). Only one of forty (2.5%) animals immunized with gD2 mRNA was positive for HSV-2 DNA in DRG obtained at the end of the experiment (Figure 5f). This animal was different from the two that had positive vaginal virus cultures on days two or four and was also in the 0.3-μg group. Overall, three of ten (30%) mice in the 0.3-μg group had evidence of subclinical infection compared to 0/30 (0%) at higher doses (*p* = 0.0121 by two-tailed Fisher’s exact test). We conclude that the gD2 mRNA-LNP vaccine provided outstanding protection against clinical disease at 1 μg; however, breakthrough subclinical infections occurred when the vaccine dose was 0.3 μg. Only the epitope-specific antibody responses offered a preview that 1 μg was likely sufficient, enabling the inclusion of higher doses of the other immunogens in the vaccine, if needed.

## 4. Discussion

We included equal concentrations of each mRNA in the gC2, gD2, and gE2 trivalent vaccine in our prior mRNA vaccine publications and the current study to define the maximum tolerated dose [4,5,6,7]. The vaccine efficacy could possibly be improved by using lower doses of one antigen and higher doses of another rather than equal doses of each antigen. The Merck nonavalent human papillomavirus vaccine (Gardasil^®^ 9) is an example of a multivalent vaccine that incorporates different doses of protein antigens in the vaccine ranging from 20 μg to 60 μg [20].

We used the murine mouse model of genital herpes to determine if epitope mapping provides useful information beyond IgG ELISA and neutralizing antibody titers during the dose escalation phase of a trial to select immunogen doses for a multivalent vaccine. We evaluated serum IgG binding assays over a 33-fold range of gD2 mRNA-LNP doses. Two immunizations were more immunogenic than one, supporting the value of IgG ELISA in selecting the dosing frequency. However, IgG ELISA was less valuable for deciding the dose per administration to include in the trivalent vaccine. A plateau in ELISA titers did not occur to guide the decision. Instead, antibody titers increased at higher doses with a significantly higher gD2 IgG ELISA titers at 10 μg than at 0.3 μg. We detected a similar dose-response pattern for neutralizing antibody titers. Toxicity studies suggested a maximum tolerated dose of 10 μg for all three mRNA-LNP immunogens. The ELISA and neutralizing antibody titers provided no clues that less than one-third of that total dose was needed for gD2 mRNA-LNP.

We used high throughput biosensor technology to evaluate antibody responses to crucial gD2 epitopes. We made four important observations. First, antibody responses to crucial gD2 epitopes plateaued at doses ≥1 μg, suggesting little benefit of using higher doses, distinguishing the epitope responses from ELISA and neutralizing antibody titers. Second, antibody responses to some gD2 epitopes were lower at 10 μg than 1 μg, indicating that using too high a dose may be detrimental from both an immunology and toxicity perspective. Third, combining antibody responses to all six crucial epitopes provided a clearer cutoff for the effective gD2 mRNA dose than analyzing individual epitopes. Fourth, the antibody responses to all six epitopes matched the results of the HSV-2 challenge studies in that antibody titers were lowest in the 0.3-μg group and this was the only group with breakthrough infections, while the 1-μg dose was as protective as the 3- or 10-μg doses.

The study has certain limitations. First, the number of breakthrough infections was small, with only 3 animals developing infection. Immunizing mice with gD2 mRNA at doses lower than 0.3 μg may yield more breakthrough infections and improve our confidence in the value of epitope mapping to predict vaccine efficacy. Nevertheless, differences in breakthrough infections were statistically significant when comparing 0.3 μg with higher doses. Second, we evaluated sera for antibodies to gD2 epitopes at a single dilution of 1:50. Results may vary if sera were tested at multiple dilutions. We think this outcome is unlikely because almost all animals produced antibodies within the dynamic range of the assay, with few animals having no antibody response or producing antibody titers at the upper limit of detection.

Subsequent studies will evaluate the optimal dose of gC2 and gE2 mRNA-LNP to include in the trivalent vaccine based on the same principles explored here for gD2 mRNA-LNP. We conclude that epitope mapping adds a valuable new tool to more traditional immunology assays for deciding the dose of each mRNA immunogen to include in a multivalent vaccine during a phase 1 dose escalation study.

## Data Availability

All the data is available within the manuscript figures and text.
